# Health literacy in a population-based sample of Australian women: a cross-sectional profile of the Geelong Osteoporosis Study

**DOI:** 10.1186/s12889-018-5751-8

**Published:** 2018-07-13

**Authors:** Sarah M. Hosking, Sharon L. Brennan-Olsen, Alison Beauchamp, Rachelle Buchbinder, Lana J. Williams, Julie A. Pasco

**Affiliations:** 1 Epi-Centre for Healthy Ageing, School of Medicine, IMPACT SRC, Deakin University, PO Box 281 (Barwon Health), Geelong, VIC 3220 Australia; 2 Australian Institute for Musculoskeletal Science (AIMSS), The University of Melbourne and Western Health, St Albans, VIC Australia; 3 Centre for Medicine Use and Safety, Faculty of Pharmacy and Pharmaceutical Sciences, Monash University, Parkville, VIC Australia; 4 Institute for Health and Ageing, Australian Catholic University, Melbourne, VIC Australia; 5 Department of Medicine-Western Health, Melbourne Medical School, The University of Melbourne, St Albans, VIC Australia; 6 Australian Health Policy Collaboration, Melbourne, VIC Australia; 7 Department of Rural Health, Monash University, Moe, VIC Australia; 8 Department of Epidemiology and Preventive Medicine, School of Public Health and Preventive Medicine, Monash University, Melbourne, VIC Australia; 9 Monash Department of Clinical Epidemiology, Cabrini Institute, Malvern, VIC Australia

**Keywords:** Health literacy, Chronic disease, Health inequities, Sociodemographic characteristics

## Abstract

**Background:**

The term health literacy refers to the abilities and resources required to find, understand and use health information in managing health. This definition is reflected in the recent development of multidimensional health literacy tools that measure multiple facets of health literacy. The aim of this study was to determine the health literacy profile of a randomly selected, population-based sample of Australian women using a multidimensional tool, the Health Literacy Questionnaire (HLQ). A second aim was to investigate associations between independent HLQ scales, sociodemographic characteristics and lifestyle and anthropometric risk factors for chronic disease.

**Methods:**

We surveyed women involved in the Geelong Osteoporosis Study (GOS), a longitudinal, population-based study. We included demographic data, lifestyle information and anthropometric measures as well as the HLQ. The HLQ has 44 items, scored on either 4- or 5-point scales, within nine conceptually distinct scales. Means for each scale were calculated, and HLQ scales were regressed on educational level and socioeconomic status. Risk factors for chronic disease were investigated using analysis of variance (ANOVA) and calculation of effect sizes.

**Results:**

Higher mean scores were seen for the scales ‘*Feeling understood and supported by healthcare professionals*’ (mean 3.20, ± SD 0.52) and ‘*Understanding health information well enough to know what to do*’ (mean 4.28, ±SD 0.54), and lower mean scores were seen for ‘*Appraisal of health information*’ (mean 2.81, ±SD 0.48) and ‘*Navigating the healthcare system*’ (mean 4.09, ± SD 0.57). Associations were also seen between lower HLQ scores and poor health behaviours including smoking and being more sedentary, in addition to greater body mass index and waist circumference. Positive gradients were seen between several HLQ scales and education level, as well as SES. For some HLQ scales, these associations were non-linear.

**Conclusions:**

The profile of this population-based cohort of women demonstrated associations between low health literacy and low SES, lower levels of education, increasing age, and anthropometric and lifestyle risk factors for chronic disease. These findings suggest implications of health literacy for health policy makers focusing on improving lifestyle prevention of chronic disease and promoting health equity at a population level.

## Background

Health literacy is defined by the World Health Organization (WHO) as ‘the cognitive and social skills which determine the motivation and ability of individuals to gain access to, understand and use information in ways which promote and maintain good health’ [1].

Previous research has estimated that 59% of Australian adults do not have adequate health literacy skills to manage their health [2]. This figure is comparable to similar high-income countries including Canada and New Zealand [3]. Within the general population there are groups more likely to report low health literacy. These include culturally and linguistically diverse (CALD) populations, individuals with lower income or education level and older adults [4–6] There is emerging evidence to suggest that low health literacy may be a mediator in the relationship between social disadvantage and poor health behaviours and outcomes [7, 8].

While the relationship between low health literacy and poorer management of chronic disease has previously been established [9–11], evidence suggests low health literacy may also influence lifestyle prevention of chronic disease. Associations between higher health literacy and some lifestyle behaviours including healthier diet and increased physical activity have been demonstrated [12–17]. However, associations between health literacy and smoking and alcohol intake have been inconsistent [12, 16, 18, 19], potentially due to the variation between study populations and health literacy measures used.

Prevention and management of chronic disease is complex and requires a broad range of health literacy abilities and supports. However, the majority of previous health literacy research has investigated only a narrow set of basic literacy and numeracy skills applied to health [20–23]. More recently, a number of multidimensional tools have been developed that enable researchers to investigate a range of abilities and contextual factors associated with health literacy [24–27]. To date, research that has employed multidimensional measures of health literacy has focused on specific populations such as university students [28, 29], recently hospitalised individuals [4, 5] and patients groups [13, 30]. There is comparatively little data investigating health literacy in the broader population.

Further research is required to understand the broad range of health literacy abilities and supports in the wider population and the role they play in the uptake of lifestyle recommendations for the prevention of chronic disease. This information would assist in informing public policy, allocating resources and developing interventions to address low health literacy and reduce health inequalities at a population level [31].

The current study aims to address gaps in the literature using a multidimensional health literacy measure, the Health Literacy Questionnaire (HLQ), in a population-based sample of women. The HLQ is a multidimensional tool developed in Australia using a grounded approach [24]. Preliminary work has shown the HLQ has acceptable measurement properties and measures health literacy across nine distinct domains [24, 32]. The HLQ is a widely used measure of health literacy, having been translated and used in many countries across the world [4–6, 29, 33]. Aside from one Danish study, which included two of the nine scales that make up the HLQ to assess health literacy in the general population [6], the full HLQ has not yet been applied to the general population.

The aim of this study was to describe the health literacy profile of a randomly recruited population-based sample of women participating in a cohort study based in south-eastern Australia. A second aim was to investigate whether there were any associations between HLQ scales and socio-demographic characteristics and risk factors for chronic disease.

## Method

### Participants

Data were collected from women participating in the prospective, population-based Geelong Osteoporosis Study (GOS); the GOS protocol has been published elsewhere [34]. In brief, a cohort of 1494 women was randomly recruited from the general population between 1993 and 1997 (77.1% participation) with a further 246 women aged 20–29 years recruited at the same time as the 10-year follow up. All participants enrolled in the GOS in December 2014 were sent the HLQ to complete, with data collection continuing until March 2016. All participants gave written, informed consent to be involved in the GOS. The Barwon Health Human Research Ethics Committee approved the study.

### Data collection

Participants completed the HLQ online or via post. Participants who required assistance in completing the questions were given the opportunity to have a friend or relative assist as well as being offered the option of completing the questionnaire over the phone with a member of the research team. A question within the HLQ itself captured information regarding whether or not participants had been assisted in completing the questionnaire and, if so, in what way they were assisted. Electronic data were collected via the Research Electronic Data Capture (REDCap) tool [35] hosted by Barwon Health, which was also used to enter and manage hard copy questionnaires.

### Measure

The HLQ is a 44-item, multidimensional tool that determines health literacy scores across nine conceptually distinct domains, each measured by an independent scale. Previous research has determined the nine scales of the HLQ measure separate health literacy constructs and have good internal consistency and reliability [24, 32].

The nine scales that comprise the HLQ are:

1. Feeling understood and supported by healthcare providers
2. Having sufficient information to manage my health
3. Actively managing my health
4. Social support for health
5. Appraisal of health information
6. Ability to actively engage with healthcare providers
7. Navigating the healthcare system
8. Ability to find good health information
9. Understand health information well enough to know what to do [24].

Each scale includes between four and six items. Scales 1–5 encompass items scored on a 4-point scale (strongly disagree, disagree, agree, and strongly agree) and reflect an individual’s supports, motivation and confidence in managing their health. Scales 6–9 are scored on a 5-point scale (cannot do, very difficult, quite difficult, easy, and very easy) and broadly capture an individual’s capability to engage with, and use health information and health services, often based on lived experiences [24].

Data, including education level, health conditions, current smoking, possession of a healthcare card (a concession card available to individuals on low-income receiving government payments), private health insurance, and physical activity level (determined by a 5-level mobility scale and analysed as ‘active’ or ‘sedentary’), were self-reported. Highest level of education was recorded as one of five different levels (‘Primary school or less’, ‘Secondary education (not completed)’, ‘Secondary education (completed)’, ‘Technical and Further Education (TAFE)/Trade’ and ‘University’). Due to small counts in the lowest education group (*n* = 28), the two lower levels of educational attainment were combined for analyses.

Alcohol consumption was determined using the Victorian Cancer Council Food Frequency Questionnaire [36] and categorised as meeting or exceeding National Health and Medical Research Council (NHMRC) of Australia guidelines of two standard drinks or less per day [37]. Height and weight were to the nearest 0.1 cm and 0.1 kg, respectively. Body mass index (BMI) was calculated as (weight in kg)/(height in metres)^2^. Waist circumference (minimal abdominal) was measured using an anthropometric tape measure and categorised as < 80 cm or ≥ 80 cm as per NHMRC guidelines [38].

Area based socioeconomic status (SES) was determined using the Australian Bureau of Statistic (ABS) Index of Relative Socio-economic Advantage and Disadvantage (IRSAD). The IRSAD is a calculation of the level of social advantage/disadvantage based on 2011 ABS census data for each ABS Census Collection District, an area that encompasses approximately 250 households. Participant residential addresses were matched with corresponding ABS collection district to determine values according to the Socio Economic Indexes For Areas (SEIFA), from which IRSAD scores were ascertained and used to categorise area-level SES into quintiles, whereby quintile 1 was the most disadvantaged and quintile 5 was the most advantaged [39].

### Analyses

Missing values for HLQ items were imputed using the expectation maximisation (EM) algorithm, as previously employed by Beauchamp et al. [4]. The EM algorithm imputes values for scales where there are no more than 2 values missing from 4 to 5 item scales and no more than 3 values missing from 6 item scales.

Effect sizes (ES) were calculated using Cohen’s d [40] for differences in mean HLQ scale scores between demographic groups; ES of 0.20 to 0.50, 0.5 to 0.80 and > 0.80 were considered small, medium, and large, respectively. For all HLQ scales, assumptions of normal distribution were not met, although responses covered the full range of each scales, with modest floor and ceiling effects. A number of scales also violated homogeneity of variances. We therefore used robust analysis of variance (ANOVA) for analysis of demographic differences in HLQ scores, using the Welch method for scales that violated homogeneity of variances.

Linear regression analyses were used to investigate associations between HLQ scale scores and SES and education level. Associations between HLQ scale scores and education level and SES quintile are presented as predicted means and *p*-values, comparing the mean scale score for categories 2–4 against the mean scale score for category 1. Post-hoc analysis was undertaken to investigate the relationship between age and alcohol intake.

Analyses were undertaken using SPSS version 22 and Minitab (version 16; Minitab, State College, PA).

## Results

Of 1032 women sent the HLQ, 20 had died, 264 could not be contacted, and 35 did not participate due to reasons including illness, age, time restraints and lack of interest. Thus, 713 women provided HLQ data and were included in this analysis. Twenty-six women were assisted to complete the questionnaire over the telephone and a further 16 women were assisted by a friend or relative.

Participant characteristics are presented in Table 1. Participant SES spanned all IRSAD levels, with similar proportions observed in the most disadvantaged (14.9%) and the most advantaged (16.9%) quintiles. Only 5 (0.71%) participants reported speaking a language other than English at home and 69 (10.4%) reported current smoking. Almost two-thirds of participants (*n* = 435) had a BMI ≥25 kg/m^2^ and 464 (70.5%) had a waist circumference of ≥80 cm.

**Table 1:** Participant characteristics (*n* = 713) given as n (%) or median (IQR)

Demographic characteristics	n (%) or median (IQR)	missing data n
Age	59.1 (45.2–70.2)	0
Lives alone	138 (19.8)	16
Secondary education incomplete	240 (33.8)	2
Education (4 levels)		2
Secondary education (incomplete)	240 (33.8)	
Secondary education (complete)	146 (20.5)	
TAFE/Trade	141 (19.8)	
University	184 (25.9)	
Private health insurance	496 (71.0)	14
Health care concession card	283 (40.5)	15
Born in Australia	614 (86.2)	1
English spoken at home	706 (99.3)	7
IRSAD Quintiles		37
1 (most disadvantaged)	101 (14.9)	
2	72 (10.7)	
3	258 (38.2)	
4	131 (19.4)	
5 (least disadvantaged)	114 (16.9)	
≥3 Health conditions	143 (20.1)	22
BMI ≥25	435 (66.3)	57
Waist circumference ≥ 80 cm	464 (70.5)	55
Sedentary activity	163 (24.5)	48
Current smoking	69 (10.4)	47
> 2 glasses alcohol per day	160 (24.1)	48

*BMI* body mass index, *IRSAD* Index of Relative Socioeconomic Advantage and Disadvantage

Mean HLQ scale scores are shown in Table 2. The highest mean score for scales 1–5 was observed for Scale 1. ‘*Feeling understood and supported by healthcare professionals*’ (mean 3.20, ± SD 0.52) while the lowest mean score was observed for Scale 5. *‘Appraisal of health information’* (mean 2.81, ± SD 0.48). Scale 9. *‘Understand health information well enough to know what to do’* displayed the highest score of scales 6–9 (mean 4.28, ± SD 0.54), while Scale 7. ‘*Navigating the healthcare system*’ displayed the lowest mean score (mean 4.09, ± SD 0.57).

**Table 2:** HLQ scores for each of the 9 scales (*n* = 712) given as mean with standard deviation (±SD), and 95% confidence interval [95% CI]

Scale	HLQ Scale	Mean (±SD) [95% CI]	Missing data (n)
		Range 1–4 (lowest - highest)	
1	Feeling understood and supported by healthcare professionals	3.20 (0.52) [3.16, 3.23]	1
2	Having sufficient information to manage my health	3.07 (0.44) [3.04, 3.11]	1
3	Actively managing my health	2.99 (0.49) [2.95, 3.02]	3
4	Social support for health	3.08 (0.50) [3.05, 3.12]	2
5	Appraisal of health information	2.81 (0.48) [2.77, 2.84]	3
		Range 1–5 (lowest - highest)	
6	Ability to actively engage with healthcare professionals	4.17 (0.58) [4.13, 4.21]	9
7	Navigating the healthcare system	4.09 (0.57) [4.05, 4.13]	10
8	Ability to find good health information	4.11 (0.59) [4.06, 4.15]	10
9	Understand health information well enough to know what to do	4.28 (0.54) [4.24, 4.32]	9

Tables 3 and 4 summarise the association between sociodemographic characteristics and anthropometric and lifestyle risk-factors, and the nine HLQ scales. ES observed for differences in mean HLQ scale scores between sociodemographic groups were all small (0.20 to 0.50). The largest ES was 0.45, which related to differences in mean scale scores for Scale 8. ‘*Ability to find good health information*’ between age < 65 vs ≥65 years (ES 0.45, 95%CI 0.40 to 0.54), self-reported health conditions < 3 vs ≥3 (ES 0.45, 95% CI 0.41 to 0.57) and also sedentary vs active physical activity (ES 0.45, 95%CI 0.40 to 0.56). Being born overseas was associated with lower mean scores in Scale 2. ‘*Having sufficient information to manage health*’ (ES 0.30, 95% CI 0.26 to 0.38) and Scale 8. ‘*Ability to find good health information*’ (ES 0.26, 95% CI 0.21 to 0.39). Having private health insurance was associated with higher mean scores in the greatest number of HLQ scales of any demographic characteristic, showing small but significant ES for all scales except Scale 5. ‘*Appraisal of health information*’. Private health insurance was also the only demographic characteristic that showed a significant ES for mean differences in Scale 1. ‘*Feeling understood and supported by healthcare providers*’ (ES 0.27, 95% CI 0.21 to 0.34).

**Table 3:** Health literacy scores by sociodemographic characteristics

		Scale 1. Feeling understood and supported by healthcare providers	Scale 2. Having sufficient information to manage my health	Scale 3. Actively managing my health	Scale 4. Social support for health	Scale 5. Appraisal of health information	Scale 6. Ability to actively engage with healthcare providers	Scale 7. Navigating the healthcare system	Scale 8. Ability to find good health information	Scale 9. Understanding health information well enough to know what to do
		Mean score (±SD)								
		Score range 1–4					Score range 1–5			
Age	< 65	3.17 (0.55) *n* = 455	**3.10 (0.44)** ***n*** **= 455**	2.98 (0.53) *n* = 454	3.07 (0.51) *n* = 455	2.83 (0.49) *n* = 455	4.18 (0.55) *n* = 451	4.11 (0.54) *n* = 451	**4.20 (0.51)** ***n*** **= 451**	**4.34 (0.48)** ***n*** **= 451**
	≥65	3.23 (0.46) *n* = 257	**3.02 (0.42)** ***n*** **= 257**	2.99 (0.40) *n* = 256	3.11 (0.48) *n* = 256	2.77 (0.47) *n* = 255	4.15 (0.61) *n* = 253	4.05 (0.64) *n* = 252	**3.94 (0.68)** ***n*** **= 252**	**4.16 (0.62)** ***n*** **= 253**
*Effect size for age (95% CI)*		*−0.12 (−0.06, −0.17)*	*0.19 (0.14, 0.24)*	*−0.02 (− 0.07, 0.03)*	*−0.08 (− 0.06, 0.13)*	*0.12 (0.08, 0.18)*	*0.05 (0.00, 0.13)*	*0.10 (0.05, 0.18)*	*0.45 (0.40, 0.54)*	*0.34 (0.29, 0.41)*
Live alone	No	3.21 (0.51) *n* = 559	3.09 (0.43) *n* = 559	2.98 (0.49) *n* = 558	3.10 (0.49) *n* = 558	2.81 (0.47) *n* = 558	4.19 (0.55) *n* = 557	4.11 (0.54) *n* = 557	**4.14 (0.56)** ***n*** **= 557**	**4.23 (0.51)** ***n*** **= 557**
	Yes	3.17 (0.56) *n* = 137	3.04 (0.48) *n* = 137	3.02 (0.44) *n* = 137	3.03 (0.53) *n* = 137	2.79 (0.53) *n* = 136	4.11 (0.67) *n* = 135	4.04 (0.69) *n* = 134	**3.99 (0.68)** ***n*** **= 134**	**4.17 (0.65)** ***n*** **= 135**
*Effect size for live alone (95% CI)*		*0.08 (0.03, 0.17)*	*0.11 (0.08, 0.19)*	*−0.08 (−0.12, −0.01)*	*0.14 (0.10, 0.23)*	*0.04 (0.00, 0.13)*	*0.14 (0.09, 0.25)*	*0.12 (0.08, 0.24)*	*0.26 (0.21, 0.37)*	*0.11 (0.07, 0.22)*
Born overseas	No	3.20 (0.53) *n* = 613	**3.09 (0.44)** ***n*** **= 613**	2.99 (0.49) *n* = 611	3.08 (0.50) *n* = 612	2.81 (0.48) *n* = 611	4.17 (0.58) *n* = 606	4.10 (0.57) *n* = 605	**4.13 (0.57)** ***n*** **= 605**	4.29 (0.53) *n* = 606
	Yes	3.20 (0.46) *n* = 98	**2.96 (0.44)** ***n*** **= 98**	2.94 (0.45) *n* = 98	3.10 (0.49) *n* = 98	2.76 (0.51) *n* = 98	4.17 (0.57) *n* = 98	4.07 (0.58) *n* = 98	**3.97 (0.69)** ***n*** **= 98**	4.20 (0.60) *n* = 98
*Effect size for live alone (95% CI)*		*0.00 (−0.04, 0.09)*	*0.30 (0.26, 0.38)*	*0.10 (0.06, 0.19)*	*−0.04 (− 0.08, 0.06)*	*0.10 (0.07, 0.20)*	*0.00 (− 0.05, 0.11)*	*0.05 (0.01, 0.17)*	*0.26 (0.21, 0.39)*	*0.17 (0.12, 0.29)*
Health care card	No	3.19 (0.54) *n* = 415	**3.13 (0.43)** ***n*** **= 415**	**3.02 (0.52)** ***n*** **= 415**	3.12 (0.48) *n* = 415	**2.84 (0.48)** ***n*** **= 414**	**4.22 (0.55)** ***n*** **= 412**	4.13 (0.54) *n* = 411	**4.22 (0.50)** ***n*** **= 411**	**4.36 (0.49)** ***n*** **= 412**
	Yes	3.22 (0.49) *n* = 282	**3.01 (0.44)** ***n*** **= 282**	**2.95 (0.43)** ***n*** **= 281**	3.05 (0.52) *n* = 281	**2.75 (0.49)** ***n*** **= 281**	**4.12 (0.60)** ***n*** **= 279**	4.06 (0.59) *n* = 279	**3.97 (0.65)** ***n*** **= 279**	**4.18 (0.57)** ***n*** **= 279**
*Effect size for HC card (95% CI)*		*−0.06 (−0.11, 0.00)*	*0.28 (0.24, 0.33)*	*0.14 (0.09, 0.19)*	*0.14 (0.10, 0.20)*	*0.19 (0.14, 0.24)*	*0.18 (0.12, 0.25)*	*0.13 (0.07, 0.19)*	*0.44 (0.39, 0.52)*	*0.34 (0.30, 0.41)*
Private health insurance	Yes	**3.24 (0.51)** ***n*** **= 495**	**3.11 (0.43)** ***n*** **= 495**	**3.02 (0.49)** ***n*** **= 495**	**3.12 (0.47)** ***n*** **= 495**	2.83 (0.48) *n* = 494	**4.22 (0.54)** ***n*** **= 490**	**4.14 (0.55)** ***n*** **= 489**	**4.15 (0.57)** ***n*** **= 489**	**4.32 (0.52)** ***n*** **= 490**
	No	**3.10 (0.54)** ***n*** **= 203**	**3.00 (0.45)** ***n*** **= 203**	**2.91 (0.48)** ***n*** **= 203**	**3.01 (0.56)** ***n*** **= 203**	2.75 (0.49) *n* = 203	**4.05 (0.64)** ***n*** **= 202**	**4.00 (0.63)** ***n*** **= 202**	**4.02 (0.63)** ***n*** **= 202**	**4.17 (0.58)** ***n*** **= 202**
*Effect size for insurance (95% CI)*		*0.27 (0.21, 0.34)*	*0.25 (0.21, 0.31)*	*0.23 (0.18, 0.29)*	*0.22 (0.18, 0.30)*	*0.17 (0.12, 0.23)*	*0.30 (0.25, 0.30)*	*0.24 (0.20, 0.33)*	*0.22 (0.17, 0.31)*	*0.28 (0.23, 0.36)*
Health conditions	< 3	3.19 (0.51) *n* = 570	**3.09 (0.41)** ***n*** **= 570**	2.99 (0.49) *n* = 568	**3.12 (0.46)** ***n*** **= 569**	2.81 (0.48) *n* = 568	**4.21 (0.54)** ***n*** **= 564**	**4.12 (0.54)** ***n*** **= 563**	**4.16 (0.54)** ***n*** **= 563**	**4.31 (0.51)** ***n*** **= 564**
	≥3	3.22 (0.57) *n* = 142	**3.00 (0.54)** ***n*** **= 142**	2.96 (0.49) *n* = 142	**2.94 (0.60)** ***n*** **= 142**	2.80 (0.51) *n* = 142	**4.02 (0.68)** ***n*** **= 140**	**3.97 (0.67)** ***n*** **= 140**	**3.90 (0.71)** ***n*** **= 140**	**4.14 (0.63)** ***n*** **= 140**
*Effect size for conditions (95% CI)*		*−0.06 (−0.10, 0.04)*	*0.21 (0.17, 0.29)*	*0.06 (0.02, 0.14)*	*0.37 (0.33, 0.47)*	*0.02 (−0.02, 0.10)*	*0.33 (0.29, 0.45)*	*0.26 (0.22, 0.38)*	*0.45 (0.41, 0.57)*	*0.32 (0.28, 0.42)*

Results in bold indicate a *p*-value < 0.05 for difference in means tested using one-way ANOVA

ES calculated using Cohen’s d. ES are interpreted as “Small” > 0.2–0.5, “Moderate” > 0.5–0.8, “Large” > 0.8

**Table 4:** Mean health literacy scores (±SD) and effect sizes (95%CI) across anthropometric and lifestyle risk factors

		Scale 1. Feeling understood and supported by healthcare providers	Scale 2. Having sufficient information to manage my health	Scale 3. Actively managing my health	Scale 4. Social support for health	Scale 5. Appraisal of health information	Scale 6. Ability to actively engage with healthcare providers	Scale 7. Navigating the healthcare system	Scale 8. Ability to find good health information	Scale 9. Understanding health information well enough to know what to do
		Mean score (±SD)								
		Score range 1–4					Score range 1–5			
BMI (kg/m^2^)	< 25	3.17 (0.53) *n* = 220	3.11 (0.41) *n* = 220	**3.10 (0.50)** ***n*** **= 219**	**3.15 (0.43)** ***n*** **= 219**	2.85 (0.48) *n* = 219	4.19 (0.54) *n* = 216	4.13 (0.54) *n* = 216	4.16 (0.54) *n* = 216	4.29 (0.52) *n* = 216
	≥25	3.23 (0.49) *n* = 435	3.07 (0.43) *n* = 435	**2.93 (0.47)** ***n*** **= 434**	**3.06 (0.51)** ***n*** **= 435**	2.79 (0.49) *n* = 435	4.19 (0.56) *n* = 432	4.10 (0.54) *n* = 431	4.11 (0.57) *n* = 431	4.29 (0.50) *n* = 432
*Effect size for BMI (95% CI)*		*− 0.12 (− 0.19, − 0.07)*	*0.09 (0.04, 0.14)*	*0.35 (0.29, 0.40)*	*0.19 (0.13, 0.23)*	*0.12 (0.06, 0.17)*	*0.00 (− 0.07, 0.05)*	*0.05 (− 0.02, 0.11)*	*0.09 (0.02, 0.14)*	*0.00 (− 0.07, 0.05)*
Waist circumference (cm)	< 80	3.19 (0.52) *n* = 194	**3.14 (0.42)** ***n*** **= 194**	**3.11 (0.53)** ***n*** **= 194**	**3.17 (0.45)** ***n*** **= 194**	2.85 (0.49) *n* = 194	4.19 (0.59) *n* = 191	4.14 (0.59) *n* = 191	**4.20 (0.59)** ***n*** **= 191**	4.33 (0.56) *n* = 191
	≥80	3.22 (0.50) *n* = 463	**3.06 (0.42)** ***n*** **= 463**	**2.93 (0.46)** ***n*** **= 461**	**3.06 (0.50)** ***n*** **= 462**	2.79 (0.48) *n* = 462	4.18 (0.54) *n* = 459	4.09 (0.53) *n* = 458	**4.08 (0.56)** ***n*** **= 458**	4.27 (0.50) *n* = 459
*Effect size for waist (95% CI)*		*−0.06 (− 0.13, − 0.01)*	*0.19 (0.13, 0.23)*	*0.37 (0.30, 0.42)*	*0.23 (0.16, 0.27)*	*0.12 (0.06, 0.17)*	*0.02 (− 0.07, 0.07)*	*0.09 (0.01, 0.14)*	*0.21 (0.13, 0.26)*	*0.12 (0.04, 0.16)*
Sedentary	No	3.20 (0.50) *n* = 502	3.09 (0.40) *n* = 502	**3.03 (0.49)** ***n*** **= 500**	3.10 (0.49) *n* = 501	2.81 (0.49) *n* = 501	**4.22 (0.54)** ***n*** **= 495**	**4.14 (0.53)** ***n*** **= 494**	**4.18 (0.52)** ***n*** **= 494**	**4.34 (0.49)** ***n*** **= 495**
	Yes	3.26 (0.52) *n* = 162	3.03 (0.52) *n* = 162	**2.87 (0.46)** ***n*** **= 162**	3.06 (0.51) *n* = 162	2.79 (0.49) *n* = 162	**4.08 (0.65)** ***n*** **= 162**	**4.00 (0.68)** ***n*** **= 162**	**3.92 (0.73)** ***n*** **= 162**	**4.11 (0.65)** ***n*** **= 162**
*Effect size for sedentary (95% CI)*		*−0.12 (−0.16, 0.04)*	*0.14 (0.10, 0.22)*	*0.33 (0.29, 0.40)*	*0.08 (0.04, 0.16)*	*0.04 (0.00, 0.12)*	*0.25 (0.20, 0.35)*	*0.25 (0.20, 0.35)*	*0.45 (0.40, 0.56)*	*0.43 (0.39, 0.53)*
Alcohol-drinks per day	≤2	3.20 (0.50) *n* = 504	**3.06 (0.43)** ***n*** **= 504**	2.97 (0.48) *n* = 503	3.07 (0.50) *n* = 503	2.80 (0.49) *n* = 503	4.17 (0.57) *n* = 498	4.08 (0.58) *n* = 497	**4.09 (0.60)** ***n*** **= 497**	**4.25 (0.55)** ***n*** **= 498**
	> 2	3.24 (0.54) *n* = 162	**3.14 (0.42)** ***n*** **= 160**	3.03 (0.53) *n* = 159	3.15 (0.46) *n* = 160	2.84 (0.48) *n* = 160	4.23 (0.51) *n* = 159	4.16 (0.50) *n* = 159	**4.20 (0.50)** ***n*** **= 159**	**4.38 (0.47)** **n = 159**
*Effect size for alcohol (95% CI)*		*−0.08 (−0.12, 0.00)*	*−0.19 (−0.22, − 0.12)*	*−0.12 (− 0.16, − 0.04)*	*−0.16 (− 0.21, − 0.09)*	*−0.08 (− 0.12, − 0.01)*	*−0.11 (− 0.16, − 0.03)*	*−0.14 (− 0.19, − 0.06)*	*−0.19 (− 0.24, − 0.11)*	*−0.24 (− 0.29, − 0.17)*
Smoking	No	3.22 (0.49) *n* = 596	3.08 (0.43) *n* = 596	**3.00 (0.48)** ***n*** **= 594**	3.10 (0.48) *n* = 595	2.81 (0.49) *n* = 595	4.17 (0.56) *n* = 590	4.09 (0.57) *n* = 590	4.11 (0.58) *n* = 589	4.27 (0.54) *n* = 589
	Yes	3.12 (0.65) *n* = 69	3.10 (0.49) *n* = 69	**2.84 (0.56)** ***n*** **= 69**	2.99 (0.58) *n* = 69	2.79 (0.51) *n* = 69	4.23 (0.66) *n* = 69	4.17 (0.59) *n* = 69	4.17 (0.68) *n* = 69	4.40 (0.52) *n* = 69
*Effect size for smoking (95% CI)*		*0.20 (0.16, 0.35)*	*−0.04 (−0.08, 0.12)*	*0.33 (0.29, 0.46)*	*0.22 (0.19, 0.36)*	*0.04 (0.00, 0.16)*	*−0.11 (− 0.15, 0.05)*	*−0.14 (− 0.19, 0.00)*	*−0.10 (− 0.15, 0.06)*	*−0.24 (− 0.29, − 0.12)*

Results in bold indicate a *p*-value < 0.05 for difference in means tested using one-way ANOVA

ES calculated using Cohen’s d. ES are interpreted as “Small” > 0.2–0.5, “Moderate” > 0.5–0.8, “Large” > 0.8

**Fig. 1:**
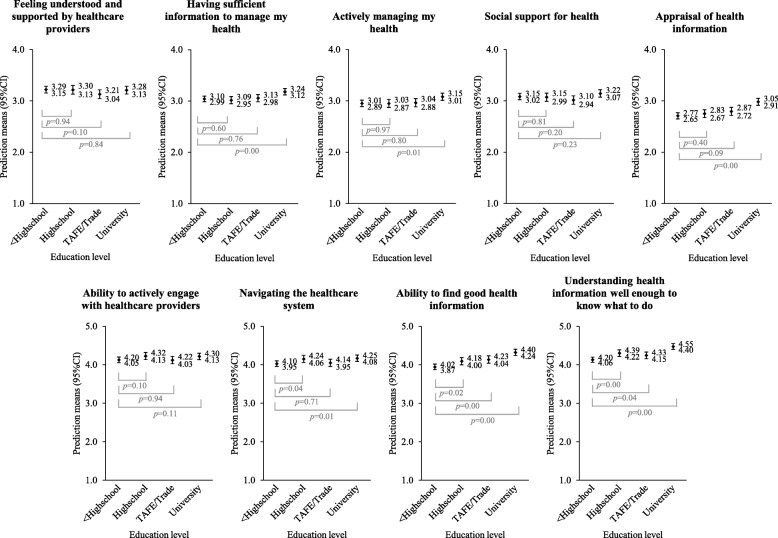
Prediction means (95% CI) for education level across health literacy scales

Figure 1 illustrates the relationship between highest self-reported education level and health literacy scales. Associations for Scale 7. ‘*Navigating the healthcare system*’ and Scale 9. ‘*Understand health information well enough to know what to do*’ were non-linear. Women who did not complete secondary education and women with a TAFE or trade qualification showed lower mean scores than individuals who self-reported their highest level of education as secondary education (complete) or a university degree. Appendix Table 6 provides *p*-values for trend across HLQ scales for both education level and SES.

**Fig. 2:**
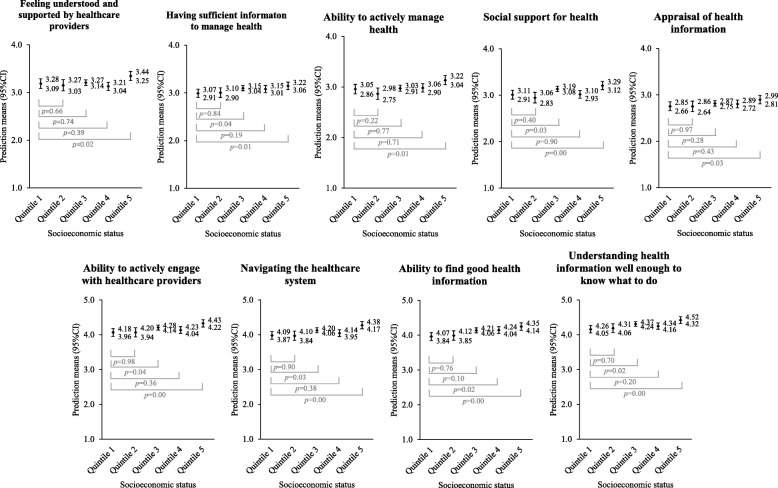
Prediction means (95% CI) for SES quintile across health literacy scales

Figure 2 describes the associations between area-level SES and seven of the nine health literacy scales. Scale 5. ‘*Appraisal of health information*’ did not show any association while Scale 2. ‘*Having sufficient information to manage health*’ showed a non-linear trend (*p* = 0.05), with SES quintiles 3 and 5 showing an association with higher scale scores while holding quintile 1 as referent. All other scales showed a significant association, however, four of these associations were also non-linear.

Table 4 shows associations between lifestyle and anthropometric risk factors for chronic disease. Having a high BMI or waist circumference were both associated with lower scores in Scale 3. ‘*Actively managing my health*’ and Scale 4. ‘*Social support for health*’. Sedentary behaviour was associated with the greatest number of health literacy scales of any lifestyle or anthropometric risk factor for chronic disease and adjusting for age, education level or SES quintile did not change associations. A high alcohol intake was associated with higher mean scores for two health literacy scales (Table 2). A post hoc analysis revealed an association between age and alcohol intake, with a greater number of women in younger age groups (including women in their 30s, 40s and 50s) more likely to consume alcohol above recommended levels than women in older age groups (data not shown).

## Discussion

Women within this study displayed strengths and difficulties across nine domains of health literacy with mean scores varying across the HLQ scales. Sociodemographic characteristics including older age, lower education level, lower area-level SES, country of birth other than Australia, and increasing number of chronic health conditions were all associated with lower health literacy. However, regression analyses revealed associations that appeared non-linear, between some HLQ scales and education level and SES quintile.

### Sociodemographic characteristics

Age and country of birth have previously shown strong associations with multiple scales of the HLQ in Australian study populations [9, 10, 41]. However, our current study showed smaller ES and associations in fewer health literacy scales for both of these sociodemographic characteristics. Specific to country of birth, it is possible that language barriers were driving the effect seen between health literacy and country of birth in earlier studies, whilst the small proportion of participants in our study that did not speak English at home (0.7%) may explain why our results differed.

Previous research has revealed inconsistent associations between health literacy and social advantage and/or disadvantage. Associations between HLQ scale scores and SES and education vary between studies, possibly related to differences in the definition or measurement of parameters of social advantage/disadvantage, for instance income, occupation, highest level of education, or having private health insurance [5, 6]. Completion of secondary education and greater number of years in education have both previously been associated with higher HLQ scores [4–6]. Our study found a similar relationship; however, we observed a non-linear relationship, with similar health literacy scores observed for the ‘TAFE/Trade’ group and the ‘Secondary education incomplete’ category. This suggests that education type, in addition to time spent in formal education or completion of secondary education, may be important to health literacy. This speculation is supported by data from university students who undertook health-based degrees and showed varying HLQ scores across the different degrees [28]. Despite the fact that all participants in that study were attending university and were therefore more likely to have higher health literacy overall, the type of degree studied was still associated with HLQ scores with the highest HLQ scores observed for medical students and the lowest for nursing students [28].

The appearance of non-linear associations with area-level SES in our study may potentially be due to the use of SES quintiles derived from IRSAD data, which provide a greater level of detail compared to other studies that employed, for instance, 2- or 3-level measures of income [5, 6]. Given that education and income variables form part of the aggregate IRSAD values, it may also be that education level, more so than income or other indicators of advantage/disadvantage, are underpinning these non-linear associations. This seems particularly likely in light of the well-documented interconnectedness between education and income, and the inextricable link between education, income and health literacy.

### Anthropometric and lifestyle risk factors

Previous research has shown a greater likelihood of lower health literacy in individuals with a chronic health condition [4, 30, 42]. This may be explained by higher health literacy needs of individuals managing a chronic disease, or low health literacy leading to chronic illness, or both. We found associations between HLQ scores and anthropometric and lifestyle risk factors known to be associated with chronic diseases, suggesting that health literacy may play a mediating role in the development of chronic disease.

The exception to this was the association between higher HLQ scores and alcohol intake above recommended levels. These associations were seen for the same HLQ scales which displayed an inverse association with age. Thus, we undertook a post-hoc analysis to determine whether age was inversely associated with alcohol intake and, in keeping with previous research [43], we observed that younger women were more likely to exceed recommendations for alcohol intake. Together these results indicate that associations between higher HLQ scores and alcohol intake above recommended levels are likely driven by age. These results are similar to a previous study of Danish adults with diabetes in which no associations were seen between alcohol consumption and the two HLQ scales assessed, Scale 9. ‘*Understanding health information well enough to know what to do*’ and Scale 6. ‘*Ability to actively engage with healthcare providers*’, after adjusting for sociodemographic characteristics, including age [12].

While, associations between levels of physical activity and HLQ scores have been seen in a small study of 36 women diagnosed with breast cancer [13], and in a large (*n* = 29,473) population-based study of Danish adults with diabetes [12], HLQ scales associated with physical activity differed across those two studies and also our current study. These differences may be due to heterogeneous study populations and use of different measures of physical activity.

### Strengths and limitations

Our study has a number of strengths. We utilised a population-based sample of women and a multidimensional measure of health literacy that enabled us to examine associations between specific aspects of health literacy and sociodemographic, anthropometric and lifestyle characteristics. The use of objective measures such as BMI and waist circumference are also a strength of this study.

A possible limitation of the study could be the underrepresentation of women with low health literacy due to the requirements of participation including the ability to read and understand the invitation to participate, complete questionnaires and attend clinical appointments. To mitigate this bias, we made efforts to offer assistance for completing questionnaires if needed. Similar to previous studies, we also avoided use of the term ‘health literacy’ in all communications to avoid the possibility that women with low literacy may have refused participation due to stigma or shame [33]. Data regarding smoking, physical activity and alcohol consumption were self-reported and are also subject to potential bias. Our current study was undertaken within a geographically defined area of regional Victoria, and thus results may not be generalisable to the wider Australian female population. Finally, this study focused on a cohort of Australian women. Future research is required to investigate whether similar associations exist in a population-based cohort of Australian men.

## Conclusion

We used a multidimensional health literacy tool to describe the health literacy profile of a randomly selected, population-based sample of Australian women and investigate associations between health literacy and sociodemographic, anthropometric and lifestyle characteristics. Mean scores varied across the nine HLQ scales indicating women in this study have strengths and difficulties in different aspects of health literacy. We report associations between lower health literacy and sociodemographic characteristics including lower SES, lower levels of education, and having been born overseas. Unlike previous studies, the associations we observed between health literacy and education and SES were non-linear, potentially due to the different measures of education and SES used. We also demonstrated associations between low health literacy and anthropometric and lifestyle risk factors for chronic disease. Further research in large population-based studies, using robust measures of lifestyle risk factors is required to better understand the relationship between lifestyle management of health and health literacy.

## Figures and Tables

**Fig. 3:**
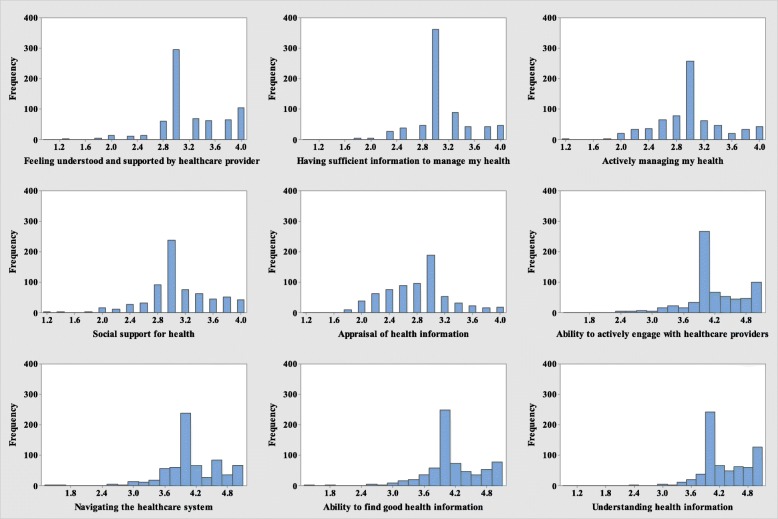
Distribution of HLQ scales

## Appendix

**Table 5:** Test of homogeneity of variances

		Scale 1. Feeling understood and supported by healthcare providers	Scale 2. Having sufficient information to manage my health	Scale 3. Actively managing my health	Scale 4. Social support for health	Scale 5. Appraisal of health information	Scale 6. Ability to actively engage with healthcare providers	Scale 7. Navigating the healthcare system	Scale 8. Ability to find good health information	Scale 9. Understanding health information well enough to know what to do
Age < 65 vs ≥65	Levene Statistic	4.20	5.33	21.06	0.61	0.01	0.05	0.03	2.29	0.68
	*p*-value	**0.04**	**0.02**	**< 0.001**	0.43	0.93	0.83	0.86	0.13	0.41
Lives alone No vs. Yes	Levene Statistic	1.28	0.01	0.19	0.33	1.80	3.31	1.61	0.11	1.35
	*p*-value	0.26	0.91	0.66	0.57	0.18	0.07	0.21	0.73	0.25
Born overseas No vs. Yes	Levene Statistic	1.32	0.28	0.09	0.47	2.20	0.07	0.00	1.71	1.09
	*p*-value	0.25	0.60	0.76	0.49	0.14	0.80	1.00	0.19	0.30
Healthcare card No vs. Yes	Levene Statistic	3.18	5.54	8.88	0.07	1.12	0.15	0.51	1.02	0.07
	*p*-value	0.07	**0.02**	**< 0.001**	0.79	0.29	0.69	0.48	0.31	0.79
Private Health Insurance No vs. Yes	Levene Statistic	1.50	7.49	0.08	0.22	0.12	0.04	0.21	0.55	0.11
	*p*-value	0.22	**0.01**	0.77	0.64	0.72	0.85	0.65	0.46	0.74
Health conditions < 3 vs. ≥3	Levene Statistic	1.52	1.19	0.14	8.48	0.35	0.85	1.78	5.42	1.52
	*p*-value	0.22	0.28	0.71	**< 0.001**	0.55	0.36	0.18	**0.02**	0.22
BMI (kg/m^2^) < 25 vs. ≥25	Levene Statistic	0.12	0.29	2.15	1.94	0.19	0.05	0.60	0.00	0.04
	*p*-value	0.73	0.59	0.14	0.16	0.66	0.82	0.44	0.95	0.84
Waist circumference (cm) < 80 vs. ≥80	Levene Statistic	0.36	3.68	6.06	0.10	0.54	2.68	2.54	1.15	1.42
	*p*-value	0.55	0.06	**0.01**	0.75	0.46	0.10	0.11	0.28	0.23
Sedentary No vs. Yes	Levene Statistic	1.13	2.52	0.00	0.03	0.13	0.17	0.18	8.60	2.03
	*p*-value	0.29	0.11	0.97	0.87	0.72	0.68	0.68	**< 0.001**	0.15
Alcohol–drinks per day < 2 vs. ≥2	Levene Statistic	2.89	2.12	4.50	0.25	0.00	0.03	0.01	0.02	0.68
	*p*-value	0.09	0.15	**0.03**	0.62	1.00	0.86	0.94	0.87	0.41
Smoking No vs. Yes	Levene Statistic	5.45	0.18	5.60	2.44	0.23	3.94	1.02	4.65	0.04
	*p*-value	**0.02**	0.67	**0.02**	0.12	0.63	0.05	0.31	**0.03**	0.84

Results in bold indicate a *p*-value < 0.05 using Levene’s test for homogeneity of variances

**Table 6:** Test for trend between education level and SES and HLQ scales

HLQ Scale	Education *p*-value	SES *p*-value
Feeling understood and supported by healthcare providers	0.37	**0.01**
Having sufficient information to manage my health	**< 0.001**	0.05
Actively managing my health	**0.03**	**< 0.001**
Social support for health	0.15	**< 0.001**
Appraisal of health information	**< 0.001**	0.19
Ability to actively engage with healthcare providers	0.18	**< 0.001**
Navigating the healthcare system	**0.04**	**< 0.001**
Ability to find good health information	**< 0.001**	**< 0.001**
Understanding health information well enough to know what to do	**< 0.001**	**< 0.001**

Results in bold indicate a *p*-value < 0.05

## Abbreviations

ABS: Australian Bureau of Statistics
ANOVA: Analysis of Variance
BMI: Body Mass Index
EM: Expectation Maximisation
ES: Effect Size
GOS: Geelong Osteoporosis Study
HLQ: Health Literacy Questionnaire
IRSAD: Index of Relative Socioeconomic Advantage and Disadvantage
NHMRC: National Health and Medical Research Council
REDCap: Research Electronic Data Capture
SD: Standard Deviation
SEIFA: Socio Economic Indexes For Areas
SES: Socioeconomic Status
WHO: World Health Organization
